# Fire and edge disturbances in the Amazon rainforest: impacts on animal–fruit and seed interactions

**DOI:** 10.1007/s00442-026-05883-9

**Published:** 2026-03-24

**Authors:** Jefferson Bruno B. S. Oliveira, Wesley Dáttilo, Hernani F. M. Oliveira, Paulo M. Brando, Walter S. de Araújo, Mathias M. Pires, Lucas N. Paolucci

**Affiliations:** 1 Programa de Pós-Graduação Em Ecologia, Departamento de Biologia Geral, Universidade Federal de Viçosa, Viçosa, MG Brazil; 2 Laboratório de Interações Ecológicas e Biodiversidade, Programa de Pós Graduação em Biodiversidade e Uso dos Recursos Naturais, Departamento de Biologia Geral, Universidade Estadual de Montes Claros, Montes Claros, MG Brazil; 3 Red de Ecoetología, Instituto de Ecología AC, Xalapa, Veracruz Mexico; 4 Laboratorio Nacional de Biología del Cambio Climático, Secretaría de Ciencia, Humanidades, Tecnología E Innovación, Mexico City, Mexico; 5 Departamento de Zoologia, Universidade Federal Do Paraná, UFPR, Curitiba, PR Brazil; 6 Yale School of the Environment, Yale University, New Haven, CT USA; 7 Instituto de Pesquisa Ambiental da Amazônia (IPAM), Brasília, Distrito Federal Brazil; 8 Instituto de Biologia, Universidade Do Estadual de Campinas, Campinas, São Paulo Brazil; 9 Departamento de Biologia Geral, Universidade Federal de Viçosa, Viçosa, Minas Gerais Brazil

**Keywords:** Ecological processes, Forest dynamics, Forest services, Forest stratification, Wildfire

## Abstract

Burned forests—particularly at their edges—are expected to lose capacity for regeneration, and subsequent disturbances may further limit it. Animal–plant interactions are critical to the resilience of tropical forests to disturbances, as they sustain key ecological functions. However, climate and land-use changes are intensifying fire regimes, potentially disrupting these interactions. Using experimental approaches with artificial fruits (in the understory and on the terrestrial level) and agricultural crop seeds (accessed by all fauna and invertebrates-only), we investigated how animal–plant interactions, focusing on key faunal groups, are affected in disturbed forests 12 years after repeated experimental fires and under continuous edge effects in the southeastern Brazilian Amazon. We found that understory animal–fruit interactions were similar across burned, edge, and undisturbed forests, whereas terrestrial animal–fruit interactions were twice as high at the unburned edge. While vertebrates accessed understory fruits more frequently, invertebrates dominated terrestrial interactions, highlighting their complementary roles. In contrast, seed interactions by both the broader faunal community and invertebrates-only declined in the interior and edge of burned forests, while invertebrate-driven seed interactions were similar between unburned edge and burned forest interior. Our findings show contrasting responses to indirect fire and edge effects across ecological interactions that support forest regeneration. Therefore, we underscore the fragility of ecological interactions through faunal access to reproductive vegetation diaspores: while animal–fruit interactions were resilient in burned forests and edges, secondary animal–seed interactions were impacted by fire. These results suggest that disturbed forests may require more than a decade to recover their interactions fully.

**Supplementary Information:**

The online version contains supplementary material available at 10.1007/s00442-026-05883-9.

## Introduction

Tropical forests are increasingly subjected to multiple human-induced pressures, primarily through habitat fragmentation and loss, which have altered the structure, conditions, and ecological functions of remaining forests (Edwards et al. 2019; Grantham et al. 2020). Approximately 180,000 km^2^ of new forest edges were created between 2001 and 2020 (Silva Junior et al. 2020; Silva et al. 2023)—and currently about 25% of tropical forests are located within 1 km of a forest edge (Taubert et al. 2018). In the Amazon, roughly 38% of the forest has already been degraded by disturbances such as severe droughts, fires, edge effects, and logging (Lapola et al. 2023; Berenguer et al. 2024), with projections indicating this figure could increase to 47% by 2050 (Flores et al. 2024). These disturbances alter microclimatic conditions at forest edges, which experience elevated temperatures, stronger wind exposure, and increased dryness. Such changes contribute to the mortality of large trees, a decline in plant diversity, and the spread of invasive grasses, especially near pastures or croplands (Silvério et al. 2019; Maracahipes-Santos et al. 2020). Together, these disturbances threaten the long-term stability and resilience of tropical (Brando et al. 2020).

Natural forest regeneration plays a vital role in mitigating the impacts of disturbances (Jakovac et al. 2021). Animal–plant interactions, driven by the manipulation of fruits and seeds, are central to forest maintenance under anthropogenic pressures that restrict or interrupt forest recovery (Selwyn et al. 2023). For example, seed dispersal by both frugivorous and secondary post-dispersal agents determines the arrival, spatial distribution, and establishment of new individuals in suitable recruitment sites (Camargo et al. 2019; Falcon et al. 2024). Conversely, although seed predation can reduce population reproductive success (Rosin and Poulsen 2018; Dylewski et al. 2020), it also acts as a selective filter by regulating competition, limiting the dominance of overly abundant species and promoting diversity (Comita et al. 2014). However, these interactions are density-dependent, with higher densities of fruits, seeds, or faunal groups promoting interactions and forest restoration (Ballarin et al. 2022). Therefore, the maintenance of animal–plant interactions is crucial for the continuity of tropical forests and the health of their ecosystem services, even under the threats posed by disturbances (Estrada-Villegas et al. 2023; Bogoni et al. 2020).

The complementary roles of different faunal groups are essential for promoting forest regeneration and enhancing ecological functions. Forest vertical stratification, for example, is key for diverse frugivore–plant interactions, as species are adapted to specific forest strata—and thus engage in distinct interactions (Schleuning et al. 2011a). Understory-feeding frugivores remove fruits from trees, while terrestrial-feeding frugivores consume fruits from lower vegetation and the forest floor, both serving as potential seed dispersers (Thiel et al. 2021; 2023). Invertebrates also play active and varied roles in diaspore dispersal, primarily at the forest floor (Pizo and Oliveira 2000; Santana et al. 2016)—although they are often overlooked in frugivory studies. Their contributions range from enhancing plant reproductive success through diplocory (Christianini and Oliveira 2010; Falcon et al. 2024) to serving as sole dispersal agents (Corlett 2021). Recognizing the complementary and overlapping roles of faunal groups across vertical gradients is therefore critical to understanding forest regeneration.

Animal–plant interactions can be abruptly disrupted when severe anthropogenic pressures reach a disturbance threshold (Batista et al. 2025; Magioli et al. 2021). For instance, fire-induced simplification of forest structure reduces critical resources for fauna, including vegetation cover and fruit and seed diversity—especially along forest edges (Cury et al. 2020; Brando et al. 2024). As a result, fire can decline faunal populations of forest specialists of both vertebrates (Barlow and Peres 2004a; b; 2006) and invertebrates (Haugaasen et al. 2003; Paolucci et al. 2017). These indirect effects of forest degradation reduce animal–plant encounters and lead to the loss of ecological interactions, including those involving frugivores (Chaves et al. 2022; Rossi et al. 2025) and secondary post-dispersal invertebrates (Paolucci et al. 2016; França et al. 2020). Driven by the long-term impacts of repeated fire on forest structure, such interaction losses can persist for years (Rossi et al. 2025), undermining the efficiency of ecological processes (Lappan et al. 2020; da Silva Rocha et al. 2022). Ultimately, disruptions to animal–plant interactions can limit natural forest recovery, thereby delaying this process (Lourenço et al. 2024).

**Fig. 1:**
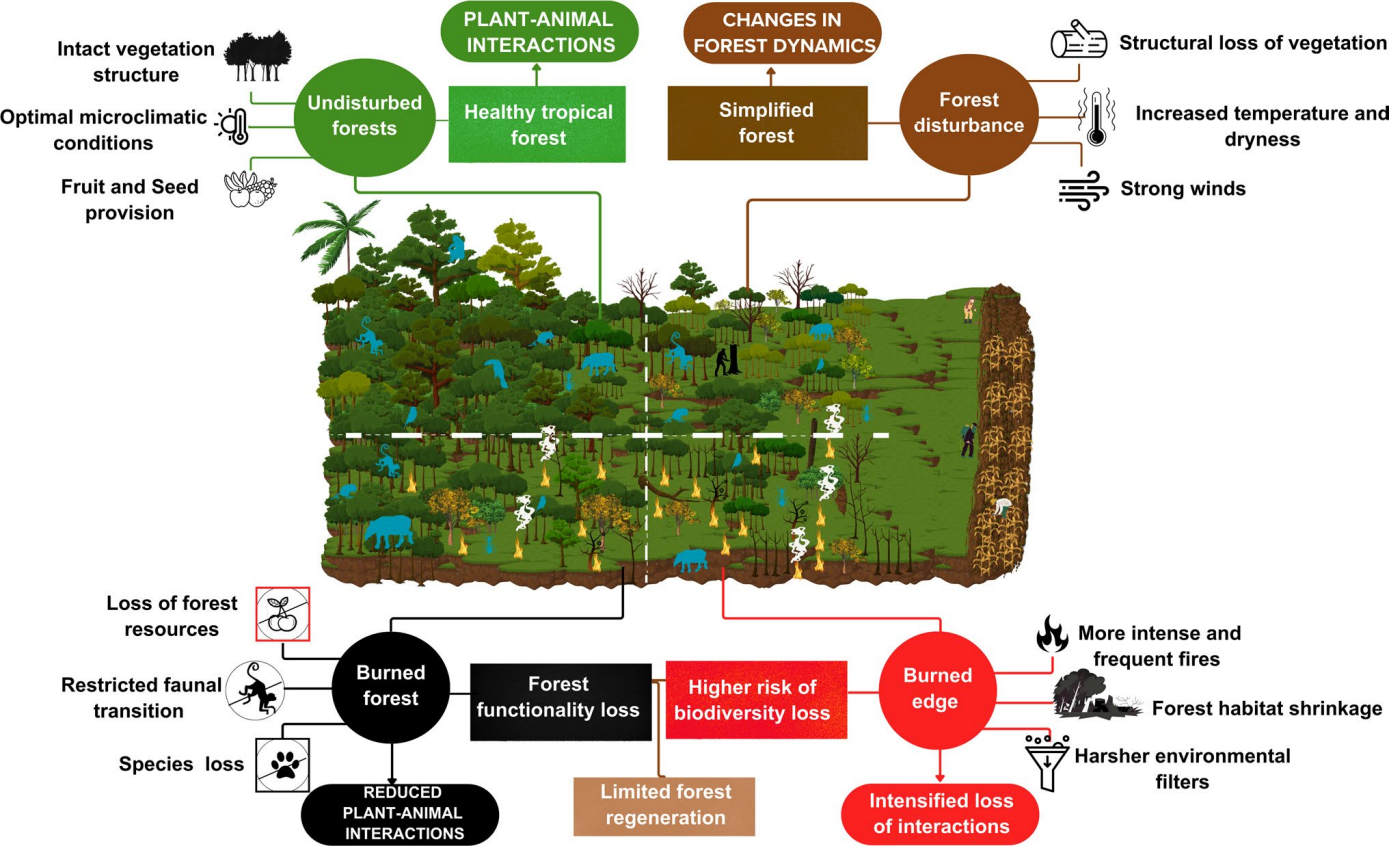
A conceptual model of the degradation processes in the Amazonian tropical forest. The model illustrates the three main disturbances and their main impacts that drive forest functionality loss, ultimately leading to limited regeneration: forest edges, fire spread, and their synergistic effects

In this study, we aimed to identify mechanisms that may slow down the natural regeneration of degraded tropical forests. Specifically, we evaluated the impacts of recurrent fires and edges on plant–animal interactions that are critical for forest recovery (Fig. 1), such as animal–fruit and animal–seed interactions. To do so, we took advantage of a long-term experiment in southeastern Amazon, characterized by limited post-fire and edge regeneration, to quantify the impacts of edges and recurrent fires on animal–plant interactions. We hypothesized that (1) fauna–fruit interactions at both the understory and terrestrial levels, and (2) fauna–seed interactions, are reduced in burned and edge forests. We expect that fruit and seed interactions occur less frequently in disturbed forests than in undisturbed ones, with the strongest reductions at burned edge. Beyond examining how interactions vary across different levels of forest degradation and vertical strata, we also assess the relative contributions of distinct animal groups to these interactions.

## Methods

### Study area

**Fig. 2:**
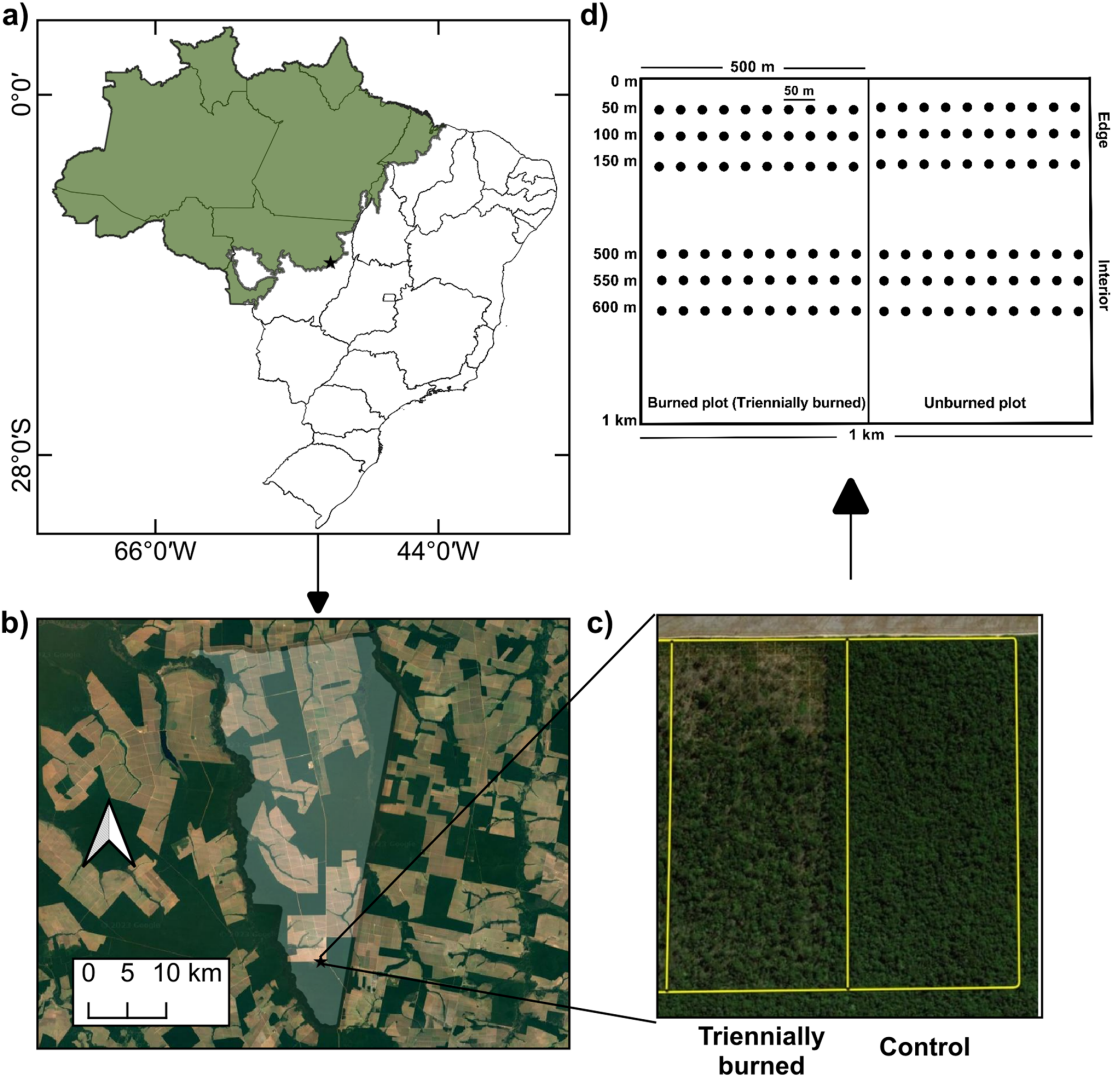
Location of the experimental area in the southeastern Amazon rainforest (Querência, Mato Grosso, Brazil): **a** Brazil, highlighting the states within the Amazon rainforest (green), with the black star representing our study area; **b** Tanguro ranch (opaque area) and its surroundings. The black star shows the experimental plots; **c** the satellite view of sampling plots; **d** a schematic representation of the sampling points within the plots burned triennially and unburned (control) and subplots (edge and interior)

The study was conducted in a southeastern Amazonian forest located in Tanguro ranch, a privately owned area (87,066 ha) in the municipality of Querência in Mato Grosso State, Brazil (13°4′27.31" S–52°22′37.34" W; Fig. 2a, b). This site lies within the “Arc of Deforestation”, one of the most active deforestation frontiers in the Amazon Basin, frequently impacted by anthropogenic fires (Marques et al. 2020; Silva et al. 2023). Approximately 60% of the ranch is composed of primary forests, with 40% undergoing land-use changes (Maracahipes-Santos et al. 2025). The forest studied has a lower canopy height (mean of 20 m) and reduced plant diversity (97 tree and liana species identified) compared to the more humid northern Amazon forests. Nine tree species account for 50% of the site’s Importance Value Index (IVI), indicating a high degree of dominance within the community (Balch et al. 2008). The experimental site edges an area initially cleared for pasture in the 80 s, which was subsequently converted to cropland in 2004 (Fig. S1). The regional climate is marked by a mean annual rainfall of 1,739 mm and a mean annual temperature between 24 and 26 ºC.

### Sampling design

Fire experiments were conducted in two paired forest plots (1 km × 500 m each) surrounded by undisturbed forest (Fig. 2b, c). One plot was experimentally burned at the end of the dry season (between September and October) in the years 2004, 2007, and 2010, after which it was left to undergo natural regeneration (hereafter triennially burned plot). The second plot remained unburned and served as a control. Before the experimental treatments, neither plot had been logged or burned. Drought events co-occurred with the fires of 2007 and 2010, leading to higher impact burnings. Additionally, the windstorms of 2012 and 2019, exacerbated by fire impacts, damaged forest structure, particularly at the edges, increasing treefall (Silvério et al. 2019). Previous studies indicated pronounced negative impacts in the triennially burned plot, including high tree mortality, reduced plant and seedling diversity, and consequently lower regeneration density, especially along the plot edges (0–200 m) (Balch et al. 2008, 2013; Cury et al. 2020). For example, the dominant tree species in the burned plot are 28% shorter and have 4% lower wood density, with these changes more pronounced at forest edges (Maracahipes et al., unpublished data). Additionally, the diversity of fruit-bearing species declined, accompanied by reduced fruit and seed production (Brando et al. 2024).

To assess the interaction between burned forests and edge effects, we divided each plot into two subplots: the forest edge (from 50 to 150 m from the forest border) and the forest interior (from 500 to 600 m from the border), so we had four distinct treatments, hereafter treated as: unburned interior (used as the control), unburned edge (representing only edge effects), burned interior (representing impacts of burned forests), and burned edge (representing the cumulative effects of burned forests and edge effects) (Fig. 2d; Fig. S1). Within each treatment, we established three sampling transects. For edge treatments, transects were positioned at 50 m, 100 m, and 150 m; for interior treatments, at 500 m, 550 m, and 600 m. Along each transect, we established 10 sampling points spaced 50 m apart, totaling 30 in each treatment. The experiments were conducted at the end of the dry season (September 2022), coinciding with the transition from the peak flowering period to the onset of fruiting in the forest.

### Animal-fruit interaction experiments

To assess the effects of burned forests and edge on fruit–animal interactions, we conducted experiments along two sampling transects per treatment: 50 m and 150 m (unburned edge and burned edge) and 500 m and 600 m (unburned interior and burned interior), with 20 sampling points per treatment. We used artificial red fruits, with a diameter of 1.5 cm, molded from non-toxic plasticine to simulate natural fruits and estimate fruit accessibility to fauna. Red plasticine fruits are effective proxies for studying frugivory patterns, as their color and size mimic traits commonly attractive to most frugivores (Duan et al. 2014; Balasa et al. 2023; Hazell et al. 2023). We established two animal–fruit interaction experiments, conducted at two vegetation strata: understory trees and the forest floor. At each sampling point, we arbitrarily selected two trees spaced 10 m apart, ensuring that no natural fruits were present within a 2 m radius to minimize confounding attractants and avoid bias from previously established frugivore visitation. We deposited 10 artificial fruits on branches approximately 1.8 m above the floor on one tree to simulate understory faunal–fruit interactions, while another set of 10 fruits was positioned at the base of a second tree to simulate terrestrial faunal–fruit interactions, using wire stalks (Fig. S2). To prevent burial by leaf litter and ensure accessibility to invertebrates, terrestrial-level fruits were elevated 3 cm above the soil surface, with wires oriented downward.

We deployed a total of 1,600 artificial fruits across the study, with 800 per stratum. For each forest treatment, we deposited 200 fruits in the understory stratum and 200 in the terrestrial stratum. Fruit exposure lasted 96 h, after which all sampling points were inspected for evidence of fruit interactions with local fauna. We classified any fruit-bearing visible marks from animals or missing fruits as an interaction event (Fig. S2 and S3). To avoid misclassification due to accidental fruit displacement, we searched the area surrounding each focal tree (within a 1 m radius). Fruits found without marks and within this radius were not considered for interaction. Multiple marks on a single fruit were recorded as a single interaction, under the assumption that they may have been caused by the same individual. To enhance our understanding of how forest disturbances influence faunal–fruit interactions, we identified the primary faunal groups responsible for interactions through their marks: birds, mammals, or invertebrates (see Fig. S3 for reference). Each fruit bearing one or more marks attributable to a specific group was counted as a single interaction event for that group. When marks from multiple groups were present on the same fruit, the interaction was attributed to all groups involved.

### Animal-seed interaction experiments

To assess the effects of burned forest and edge on animal–seed interactions, we conducted experiments along three transects per treatment: 50 m, 100 m, and 150 m (unburned edge and burned edge), and 500 m, 550 m, and 600 m (unburned interior and burned interior), totaling 30 sampling points per treatment (Fig. 2d). We used hulled sunflower seeds (*Helianthus annuus*), a non-native agricultural crop species, as a standardized resource. Each sampling point received 10 seeds (mean ± SD: height = 7.95 ± 0.70 mm; weight = 0.04 ± 0.01 g). Sunflower seeds are nutritious and lack significant chemical or physical defenses, making them broadly attractive to a wide range of granivores and promoting consistent predation behavior. This choice minimized potential biases associated with native seeds, which may elicit specialized responses from local seed predators due to prior exposure or learned foraging behaviors within specific treatment plots (e.g., Christianini and Galetti 2007; Hargreaves et al. 2024). We used a batch of organic seed purchased from the same supplier to ensure seed consistency. Only undamaged seeds were selected so that we could attribute any observed damage to granivores. To prevent germination, we sterilized the seeds in an oven at 110 °C for 1 h.

To evaluate animal–seed interactions across different faunal groups, we established two seed depots at each sampling point. One depot contained 10 seeds fully accessible to all fauna (hereafter full-fauna access). At the same time, the second depot, also with 10 seeds, was enclosed in a conical metal cage (12 cm high, 15 cm in diameter, and a mesh size of 2.5 × 2.5 cm), anchored to the floor using metal hooks: this cage design excluded vertebrates while permitting access to invertebrates (hereafter, invertebrates-only access). Both depots were located 1 m apart on previously cleared soil (Fig. S4). This experimental setup enabled us to evaluate seed interactions involving the entire fauna while isolating the specific contributions of invertebrates.

We deployed a total of 2,400 seeds across the experiments, with 1,200 assigned to full-fauna access and 1,200 to invertebrates-only access. Each forest treatment included 300 seeds per experimental access. We inspected each depot 24 h after seed deposition to assess interactions with fauna. As the lipid-rich sunflower seeds used in this study lack other structural features that might influence consumption or dispersal behavior, most seeds were likely predated rather than dispersed. However, interpreting missing seeds solely as evidence of predation or dispersal, without tracking their fate, can be biased (see Vander Wall et al. 2005; Penn and Crist 2018). Therefore, we treated these seeds as standardized models to assess the impacts of forest disturbances on animal–seed interactions (seed manipulation—following Fernandes et al. 2018), regardless of the seeds’ ultimate fate. We recorded all events indicating seed interactions by fauna, defined as either visible consumption marks or complete seed removal from the depot. For descriptive purposes, seeds bearing consumption marks were classified as predated, while missing seeds were categorized as removed.

### Statistical analysis

To investigate the effects of burned forests and edges on animal–plant interactions, we calculated the proportion of diaspores manipulated (i.e., the number of fruits or seeds with signs of interactions divided by the total number of available fruits or seeds). We conducted these analyses using generalized linear models (GLMs). For animal–fruit analysis, we built two separate models to evaluate the impact on each vegetation strata independently: one for understory fruit interactions and another for terrestrial fruit interactions. Similarly, we created two models for seed interactions: one for the full-fauna access and another for the invertebrate-only access. In all models, the response variables were the proportion of fruit or seed interactions. The predictor variable was forest treatment type, categorized into four levels: unburned interior (control), unburned edge (edge effects), burned interior (burned forest), and burned edge (fire-edge synergy). We used a binomial error distribution and applied corrections for overdispersion through a beta-binomial distribution using the *glmmTMB* package (Brooks et al. 2017). We analyzed the residuals to check the distribution’s adequacy and the models’ fit using the *DHARMa* package (Hartig 2022). The significance of the models was tested using type II Anova from the *car* package (Fox and Weisberg 2019). When models were significant, we performed contrast tests with pairwise comparisons separately, combining treatment categories within models.

## Results

**Fig. 3:**
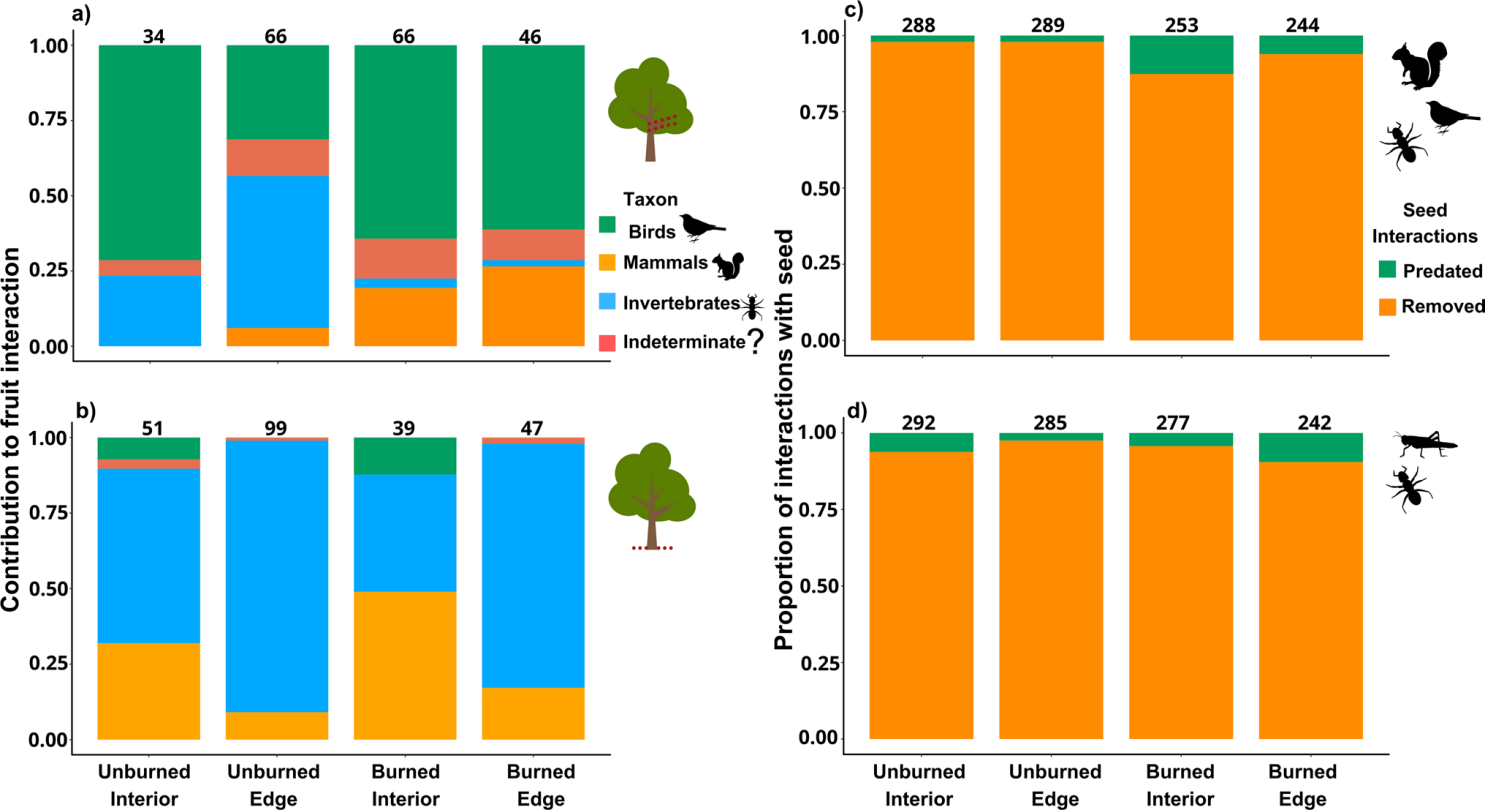
The relative contribution of fauna to **an** understory and **b** terrestrial artificial fruit interaction by fauna and the relative seed interaction for **c** full-fauna access and **d** invertebrates-only access. The values are based on total interactions across unburned interior, unburned edge, burned interior and burned edge treatments. In animal–fruit interactions, faunal groups include birds (green), indeterminate (salmon), invertebrates (red), and mammals (yellow). In animal–seed interactions, include predated seeds (seen with predation marks; green) and removed seeds (missing; orange). The number of interactions with fruits and seeds is above the bars

**Fig. 4:**
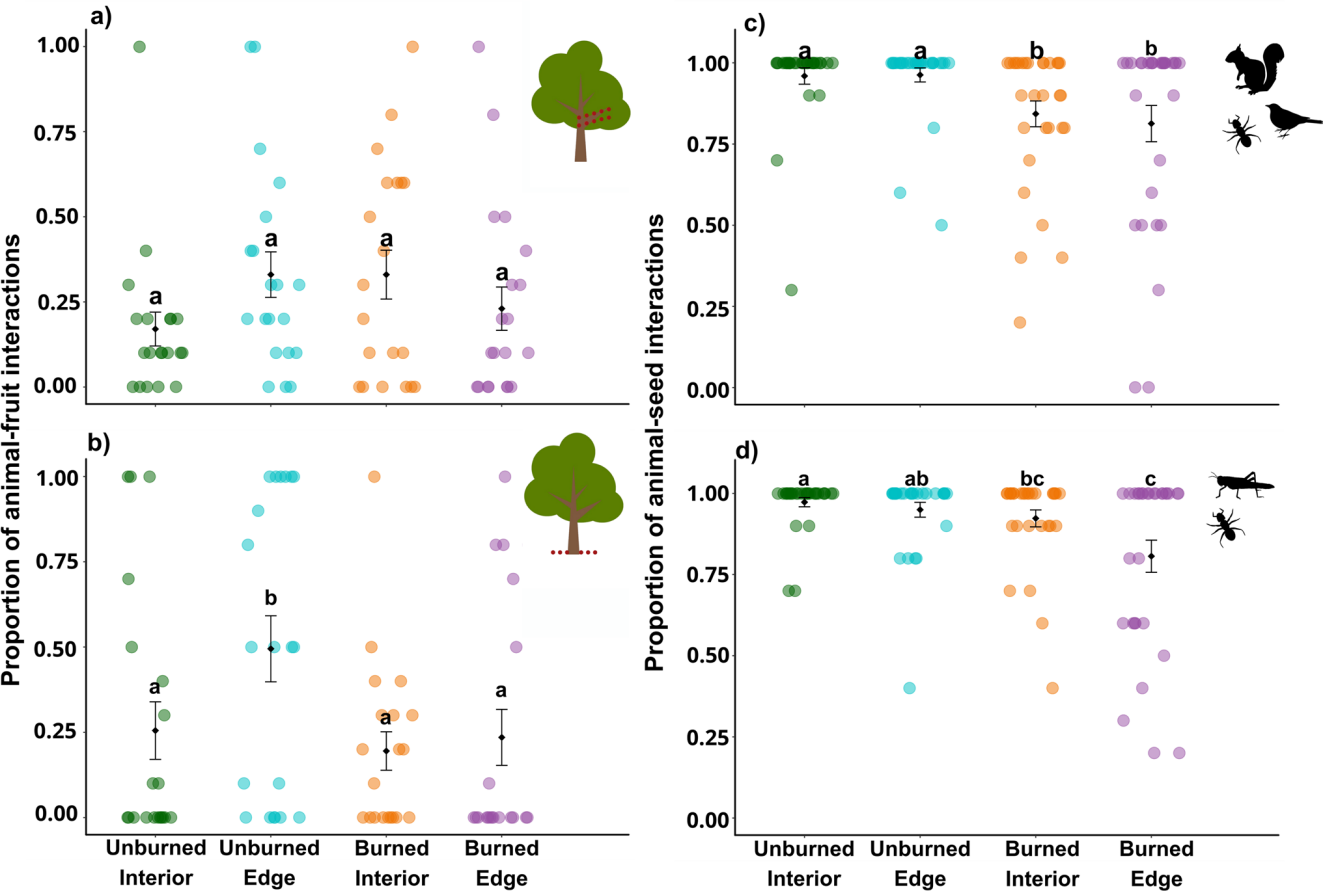
Mean proportion of interactions with artificial fruits and seeds by fauna across unburned interior, unburned edge, burned interior, and burned edge treatments. The animal–fruit experiments were conducted **a** in the understory stratum and **b** in the terrestrial stratum. The animal–seed experiments were conducted between full-fauna access **c** and the invertebrates-only access **d**. Different letters represent significant differences at alpha = 5% by post hoc groupings and do not imply an ordered ranking among treatments. Black diamonds represent mean values. Bars represent standard errors (SEs). Colored dots indicate sample points. Some samples are not visible due to overlapping

Animal–fruit interactions were widespread among forest treatments and involved a variety of animal groups (Fig. 3a; b). Most fruits were bitten by invertebrates, followed by birds and mammals (Table S1). However, the relative importance of each group varied by stratum. In the understory, birds were the primary agents (54.2%), followed by invertebrates (21.2%) and mammals (13.6%). At the terrestrial level, invertebrates interacted the most (75.8%), followed by mammals (22.93%) and birds (3.8%). Among invertebrates, ants were the most frequent fruit biters across both strata, accounting for 73.33% of invertebrate interactions in the understory stratum and 91.06% at the terrestrial stratum. Animal–fruit interactions varied widely across treatments, ranging from zero to 100% in both understory and terrestrial strata (Fig. 4a; b; Table S2). In the understory, the proportion of animal–fruit interactions was similar among treatments (Chi = 4.94; Df = 3; *P* = 0.17). However, at the terrestrial stratum, animal–fruit interactions in the unburned edge were nearly twice as large as those in the other treatments, which were similar to each other (Chi = 7.41; Df = 3; *P* = 0.05), (Table S3).

Animal–seed interactions also varied widely across forest treatments, ranging from 0 to 96% under full-faunal access and from 2 to 97% under invertebrate-only access, with similar overall interaction rates (89.5% and 91.3%, respectively) (Fig. 3c, d; Table S4, S5). Overall, most seeds were removed, and only a few were classified as predated (Fig. 3c, d; Table S4). Forest disturbance affected animal–seed interactions in both the full-fauna access (Chi = 14.86, Df = 3; *P* < 0.002) and the invertebrates-only access (Chi = 9.34, df = 3; p = 0.02). In full-fauna access, animal–seed interactions were about 16% higher in unburned interior and unburned edge than in burned interior and burned edge (Fig. 4c), with no differences between unburned interior and unburned edge or between burned interior and burned edge. Similarly, in invertebrates-only access, seed interactions were 5.42% higher in the unburned interior than in the burned interior and 20% higher than in the burned edge. Additionally, the unburned edge exhibited 17% more invertebrate–seed interactions than the burned edge—showing a marked negative effect of fire on animal–seed interactions (Fig. 4d; Table S6).

## Discussion

The resilience of tropical forests to fire and edge effects—two of the most pervasive disturbances in these habitats—is critical to their long-term persistence in the face of escalating land-use pressures. Although animal–plant interactions are fundamental to forest regeneration processes, they are sensitive to disturbances (Bello et al. 2015), and relatively few studies have been conducted in tropical forests compared with other ecosystems, thereby leaving major knowledge gaps (Ballarin et al. 2024). We found that understory fauna–fruit interactions in our studied forests, primarily driven by birds, were similar across unburned interior, unburned edge, burned interior, and burned edge. In contrast, terrestrial faunal–fruit interactions, largely driven by invertebrates, increased at the unburned edge compared to all the other treatments. Seed interaction in full-fauna access declined in burned interior and burned edge compared to unburned interior and edge. However, despite also declining in burned interior and burned edge, the interactions with seeds in invertebrates-only access were similar between the unburned edge and the burned interior, indicating complex responses among faunal groups to disturbance gradients. Because multiple faunal groups interact synergistically to sustain animal–plant interactions, our results show subtle yet persistent shifts in these ecological interactions within an Amazonian forest, persisting long after fire events and under ongoing edge influence.

Contrary to our first hypothesis, fauna–fruit interactions in the understory were not reduced in disturbed forests by either fire or edge effects. Notably, terrestrial interactions were twice as frequent at unburned edge compared to other treatments. Although species-specific interaction losses in frugivory are often expected following deforestation or fire in tropical forests (Lee et al. 2022; Rossi et al. 2025), our results show that interaction frequency—at least in the understory—can persist despite disturbances (e.g., Schleuning et al. 2011b; Malhi et al. 2022). Accordingly, previous research in our study area documented increased bird predation on model caterpillars 8 years after fire (Queiroz et al. 2022), suggesting a degree of resilience in certain ecological interactions within burned forests. Such interaction resilience—even though it can lead to lower functional quality of ecological interactions, is often derived from opportunistic and generalist species that dominate post-fire communities across vertebrates (Barlow and Peres 2004b; Rossi et al. 2025) and invertebrates (Paolucci et al. 2016; 2017). However, as higher animal–fruit interaction frequency can be a crucial pathway for frugivory and seed dispersal (Campagnoli et al. 2024), our findings underscore the persistence of key animal–plant interactions with potential implications for forest regeneration in forests burned a decade ago or exposed to constant edge effects.

Contrary to our second hypothesis, animal–seed interactions with both the broader faunal community and invertebrates alone decreased only across the burned forests (interior and edge), but remained similar at the unburned edge compared to the unburned interior. Our findings are consistent with previous research in a nearby forest, which documented reduced seed removal by ants shortly after fires (Paolucci et al. 2016), but they contrast with patterns observed in fire-adapted ecosystems, where resilient dispersal agents often increase animal–seed interactions following fire (e.g., Andersen 1988; Parr et al. 2007; Alcolea et al. 2022). These results indicate that the cumulative impacts of burned forest exert a stronger negative effect on secondary interactions than edge effects alone. These impacts highlight the fragility of secondary agents and their interactions in burned tropical forests for even 12 years after fires cease, thereby constraining their ecological interactions.

Intriguingly, burned edge did not reduce the interaction frequency of animals with either fruits or seeds. Fire induces abiotic changes similar to those observed at forest edges, including elevated temperatures, increased wind exposure, reduced moisture, and the replacement of climax vegetation by pioneer species (Silvério et al. 2019). These conditions can facilitate the invasion of exotic plants, effectively transforming burned forest interiors into extended edge environments (Brando et al. 2019; Silvério et al. 2019). Yet, our findings suggest that while edge effects can intensify fire impacts in tropical forests (Balch et al. 2008; 2009; 2015), such amplification does not reduce the frequency of animal–fruit or seed interactions. However, vegetation opening in disturbed tropical forests affects species activity and distribution, including ants, with an influx of generalist species and a loss of forest specialists (Paolucci et al. 2017). Taken together these results place the forest edge at an intermediate level of impact between undisturbed and burned forests, which helps to explain the comparable levels of animal–seed interactions observed between the unburned edge and the burned interior in the invertebrate-only access (see Andersen 2019). Curiously, the increased animal–fruit interactions on the terrestrial level observed at unburned edge may reflect the resilience of ants accessing fruit on the forest floor, likely generalist species favored by edge-related conditions.

Beyond the collaborative contributions of multiple faunal groups to interactions, we observed clear stratification in animal–fruit interactions. Vertebrates—primarily birds—accounted for most understory interactions (67.92%), whereas invertebrates—mainly ants—dominated terrestrial interactions (75.84%; Table S1). Additionally, animal–seed interaction rates were similar between full-faunal and invertebrates-only treatments (89.5% and 91.3%, respectively; Table S4). This result indicates that vertebrate access did not increase animal–seed interactions and that invertebrates—particularly ants—were the primary post-dispersal agents across all forest treatments. Ants actively forage for diaspores on the forest floor, contributing to both primary and secondary seed dispersal, as well as seed predation (Passos and Oliveira 2003; 2004; Rico-Gray and Oliveira 2007). In addition, generalist ant species, which often dominate post-fire forest communities (Paolucci et al. 2017), explore a wide range of resources in disturbed forests, particularly along edges (Meyer et al. 2009; Gerhold et al. 2019). In some cases, they may even outcompete vertebrates for access to resources in burned forests (Rossi et al. 2022). These findings, along with our results, highlight complementary patterns of vertebrates and invertebrates across vegetation strata—vertebrates at the understory and ants at the terrestrial level—which are crucial for sustaining ecological interactions and functions such as frugivory and vegetation dispersion (e.g., Pizo et al. 2005; Christianini and Oliveira 2009, 2010; Camargo et al. 2016). Although we cannot assess whether interactions with vertebrates were lost in burned forest or forest edge, the dominant role of ants and other invertebrates in mediating primary and secondary animal–plant interactions suggests they may partially act under the absence of vertebrate-mediated interactions, particularly in lower forest strata (see Rico-Gray and Oliveira 2007; Griffiths et al. 2018; Anjos et al. 2020). We therefore emphasize that the full spectrum of faunal–plant interactions—especially those involving invertebrates—is essential to conservation strategies.

Animal–seed interactions can generate contrasting but complementary functions in forest regeneration. First, seed predation represents a major constraint on plant germination, whereas dispersal is the main mechanism promoting plant recruitment. Thus, the observed reductions in seed interactions may alter vegetation assembly and structure by the reduced seed predation and dispersal (Culot et al. 2017; Larios et al. 2017; Fiedler et al. 2021). Although seed predation can reduce plant populations, reduced seed predation may favor overpopulation of pioneer or invasive species, commonly in disturbed habitats, increasing competition and structural homogenization (Paine & Beck 2007; Terborgh 2012). Additionally, reduced seed dispersal may hinder the colonization of key species, thereby reducing diversity and compromising important ecological functions, such as carbon sequestration (Fricke et al. 2025a, b). Despite their dominance in removing terrestrial-level diaspores, invertebrate-mediated interactions cannot fully substitute vertebrate interactions: invertebrates are constrained by diaspore size (Pizo and Oliveira 2001; Christianini et al. 2012; Anjos et al. 2018), and often facilitate the recruitment of exotic or pioneer plant species (Fernandes et al. 2020; Pereyra et al. 2022) (Fig. S5g). In contrast, vertebrates disperse a wide variety of seeds over long distances, frequently promoting germination by breaking dormancy and enriching soil with nutrients through their feces (Wandrag et al. 2015; Camargo et al. 2019; Soltani et al. 2018), particularly large-bodied ones (Fragoso and Huffman 2000; Paolucci et al. 2019). The limited regeneration of the disturbed forests at the time of our study is in part due to the dominance of exotic grass and pioneer species such as *Mabea fistulifera* (see Balch et al. 2008; Silvério et al. 2013; Brando et al. 2019, 2024)—a myrmecochorous plant commonly found at forest edges (Fernandes et al. 2020; Fig. S5d; g). Therefore, the decreased secondary animal–seed interactions we observed in burned forests (interior and edge), as well as invertebrates’ dominance of diaspores at the terrestrial level, can directly contribute to this restricted plant regeneration and warrant future consideration.

In conclusion, we found that animal–fruit or seed interactions across strata respond differently to burned forests and edge effects. These findings underscore the complex nature of critical interactions to forest regeneration and the dynamics of tropical forests under anthropogenic pressures. Vertebrates and invertebrates contributed in complementary ways to these interactions, with birds dominating the understory fruit access and invertebrates, mainly ants, playing a central role in forest floor diaspore interactions. The fauna can maintain access frequency to available fruits despite anthropogenic disturbances, such as 1-decade-old burned forests or constant edge effects, as we did not observe a reduction in animal–fruit interactions in the unburned edge, burned interior, or burned edge compared to the unburned interior (intact forest). In contrast, the access to seeds is fragile to perturbations mediated by fire. Ultimately, although faunal fruit access was resilient, these forests may require more than a decade to fully recover their ecological interactions, particularly secondary animal–seed interactions. In the face of ongoing degradation of tropical forests, our findings underscore faunal access to reproductive vegetation diaspores—fruits or seeds—as an ecological mechanism prone to impacts that must be protected in disturbed forests undergoing natural regeneration. Supporting stronger policies aimed at maintaining and restoring animal–plant interactions across different faunal groups, including invertebrates, can be fundamental in disturbed forests.

## Supplementary Information

Below is the link to the electronic supplementary material.

Supplementary file1 (PDF 958 KB)

## Data Availability

The datasets used and/or analyzed during the current study are available from the corresponding author on reasonable request.
