# Supplementary material for: Fire and edge disturbances in the Amazon rainforest: impacts on animal–fruit and seed interactions
Source: Oecologia. 2026 Mar 24;208(4):46. doi: 10.1007/s00442-026-05883-9 (PMC13013192; doi:10.1007/s00442-026-05883-9)
Supplement: Supplementary file 1 — Supplementary file1 (PDF 958 KB) [file 442_2026_5883_MOESM1_ESM.pdf]

Fire and edge disturbances in the Amazon rainforest: impacts on animal-fruit and seed interactions

Jefferson Bruno B. S. Oliveira[1, 2], Wesley Dáttilo[3, 4], Hernani F. M. Oliveira[5], Paulo M. Brando[6, 7], Walter S. de Araújo[2], Mathias M. Pires[8], Lucas N. Paolucci[9]

[1] Programa de Pós-Graduação em Ecologia, Departamento de Biologia Geral, Universidade Federal de Viçosa, Viçosa, MG, Brazil

[2] Laboratório de Interações Ecológicas e Biodiversidade, Departamento de Biologia Geral, Universidade Estadual de Montes Claros, MG, Brazil

[3] Red de Ecoetología, Instituto de Ecología AC, Xalapa, Veracruz, Mexico

[4] Laboratorio Nacional de Biología del Cambio Climático, Secretaría de Ciencia, Humanidades, Tecnología e Innovación, Mexico City, Mexico

[5] Departamento de Zoologia, Universidade Federal do Paraná, UFPR, Curitiba, PR, Brazil

[6] Yale School of the Environment, Yale University, New Haven, Connecticut, USA

[7] Instituto de Pesquisa Ambiental da Amazônia (IPAM), Brasília, Distrito Federal, Brazil

[8] Instituto de Biologia, Universidade do Estado de Campinas, Campinas, São Paulo, Brazil

[9] Departamento de Biologia Geral, Universidade Federal de Viçosa, Viçosa, Minas Gerais, Brazil

Supplementary information

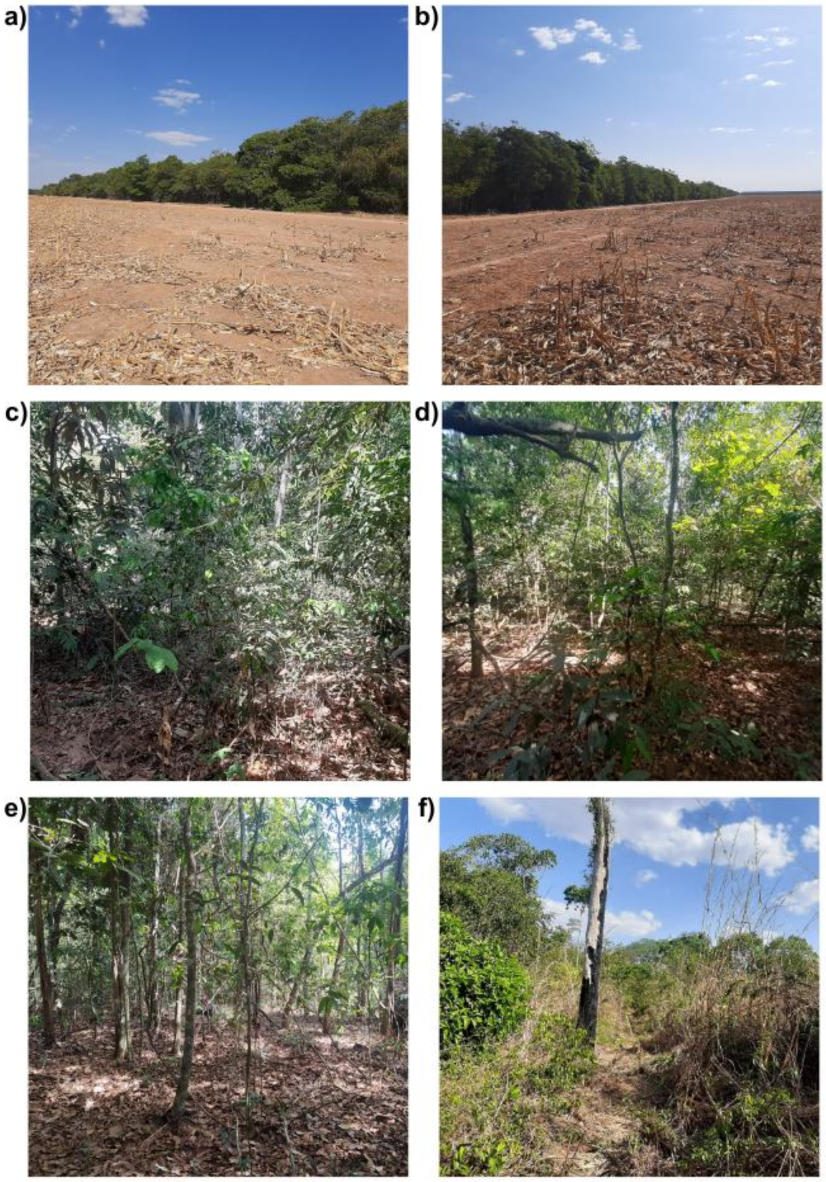

Fig. 1 Forest areas studied 12 years after a controlled fire experiment: **a-b)** transition of the unburned and burned forest with the agricultural cultivation area, respectively; **c)** unburned interior; **d)** burned interior; **e)** unburned edge; **f)** burned edge

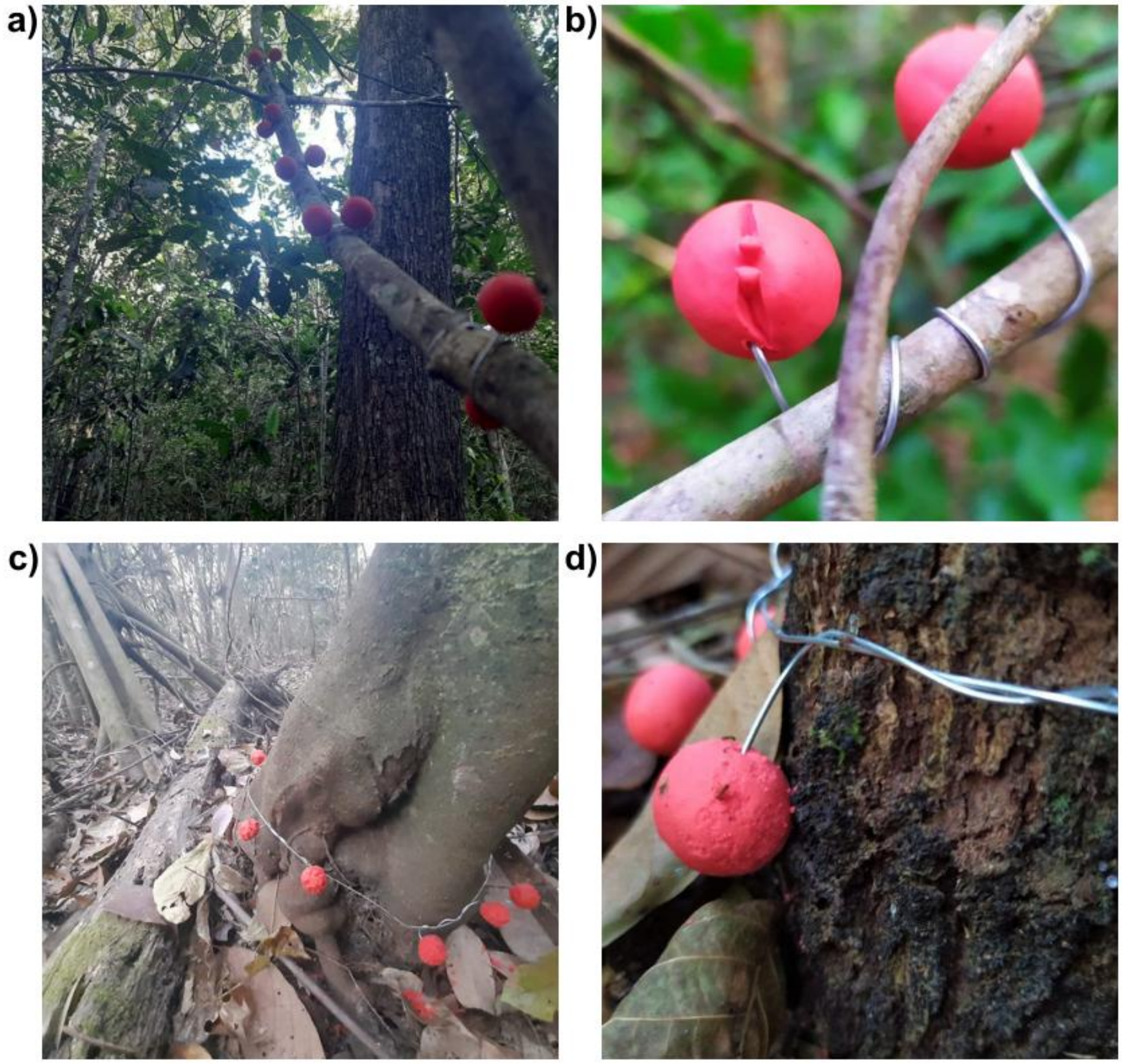

Fig. 2 Artificial fruits deposited to evaluate the establishment of animal-plant interactions carried out by fauna: **a)** artificial fruits deposited on branches (understory); **b)** marks made by birds; **c-d)** artificial fruits deposited on the ground with marks of invertebrates (ants)

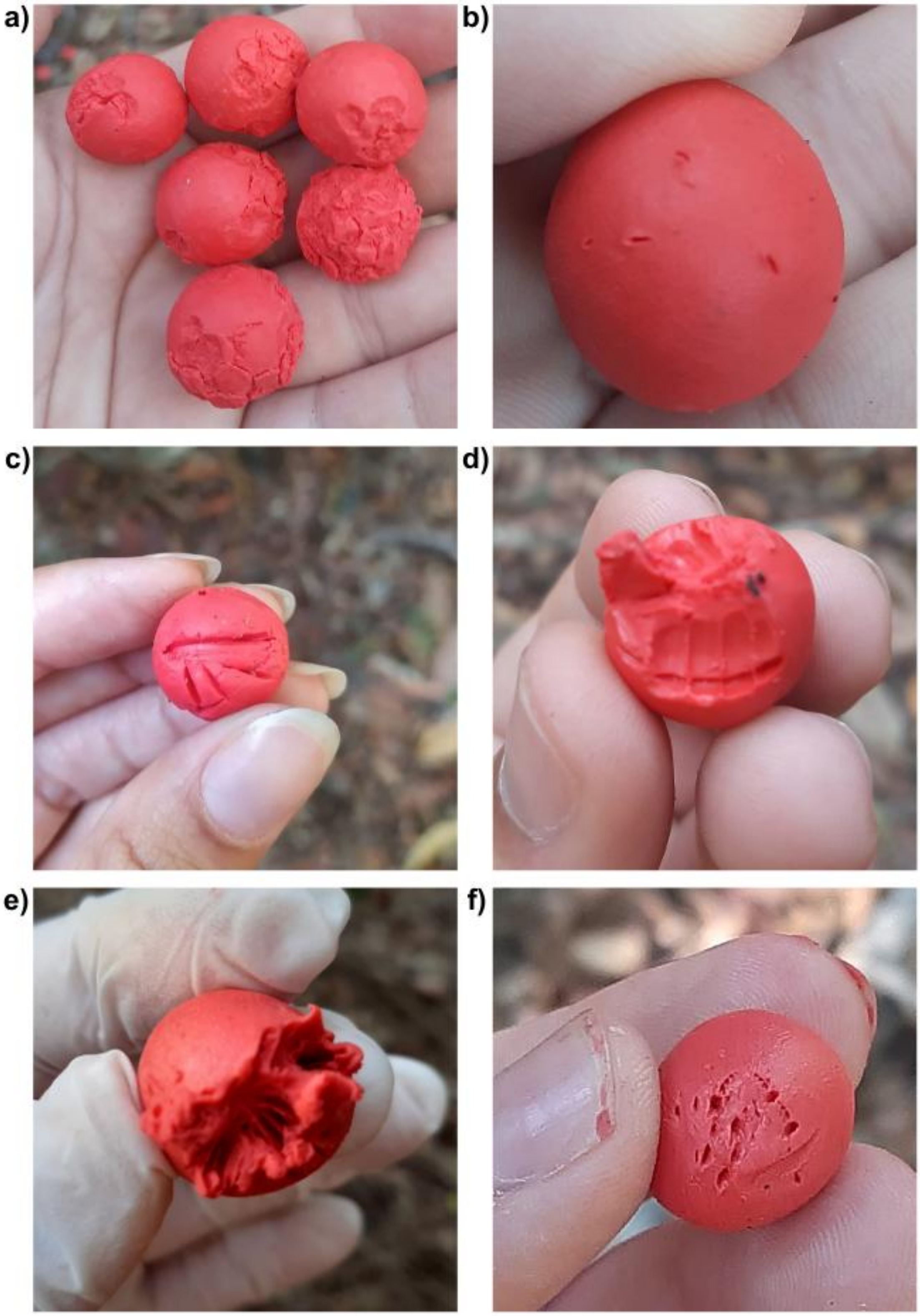

Fig. 3 Artificial fruits with the marks of different faunal groups: **a-b)** invertebrates (ants and other invertebrates, respectively); **c)** birds; **d-f)** mammals (primates, rodents and marsupials, respectively). Birds: triangular marks or peck marks; mammals: bite marks

with teeth; invertebrates: ants leave circular cut marks or small perforations, while other invertebrates leave small scraping marks or two converging scratches

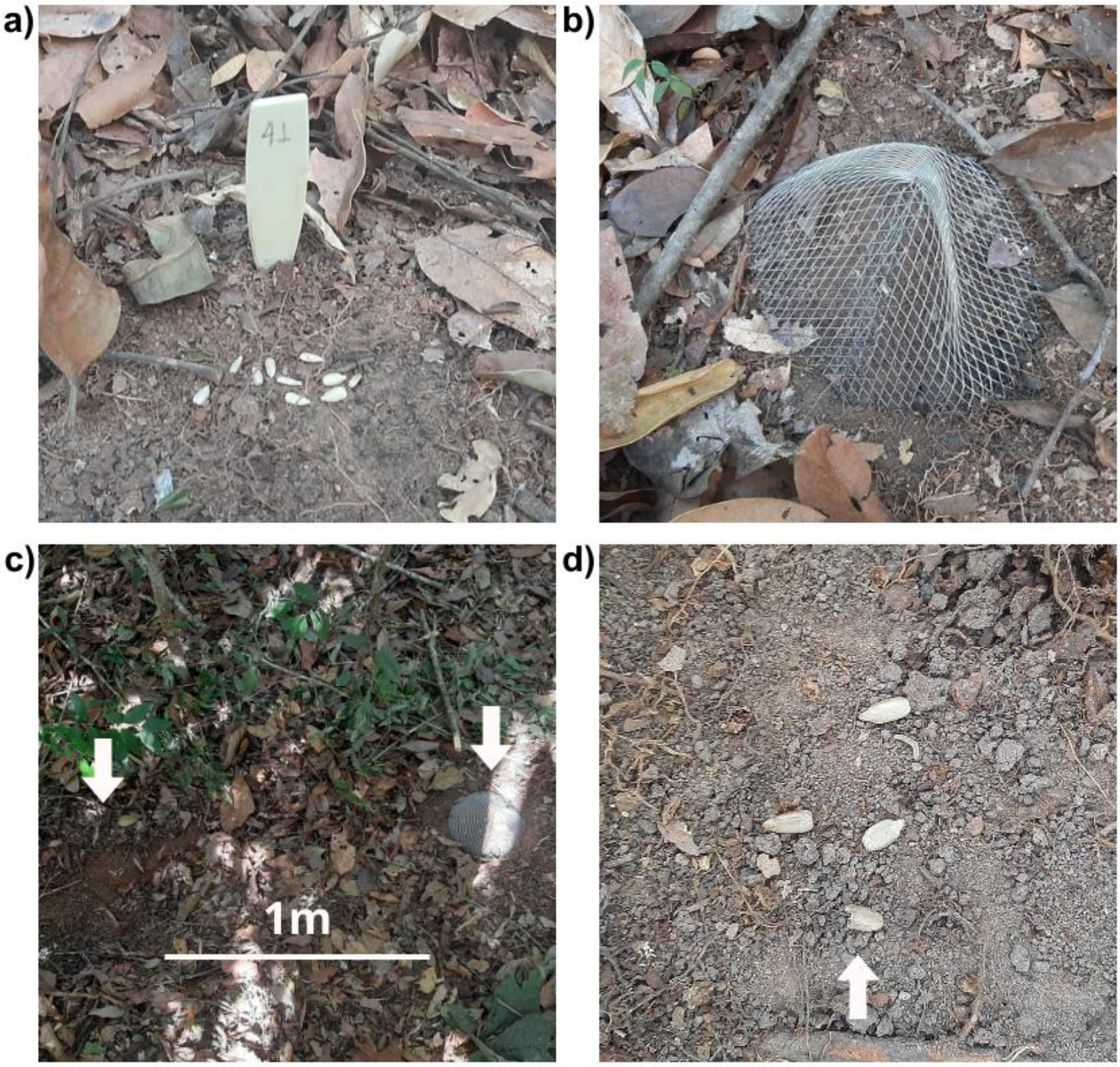

Fig. 4 The seed deposition in the field: **a)** seeds deposited in the experiment without a cage for free access by all groups; **b)** seeds deposited using cages to exclude vertebrates and allow exclusive access by invertebrates; **c)** demonstration of seed deposition with and without cages at each sampling point; **d)** seeds remaining after interaction with animals with seeds showing predation marks

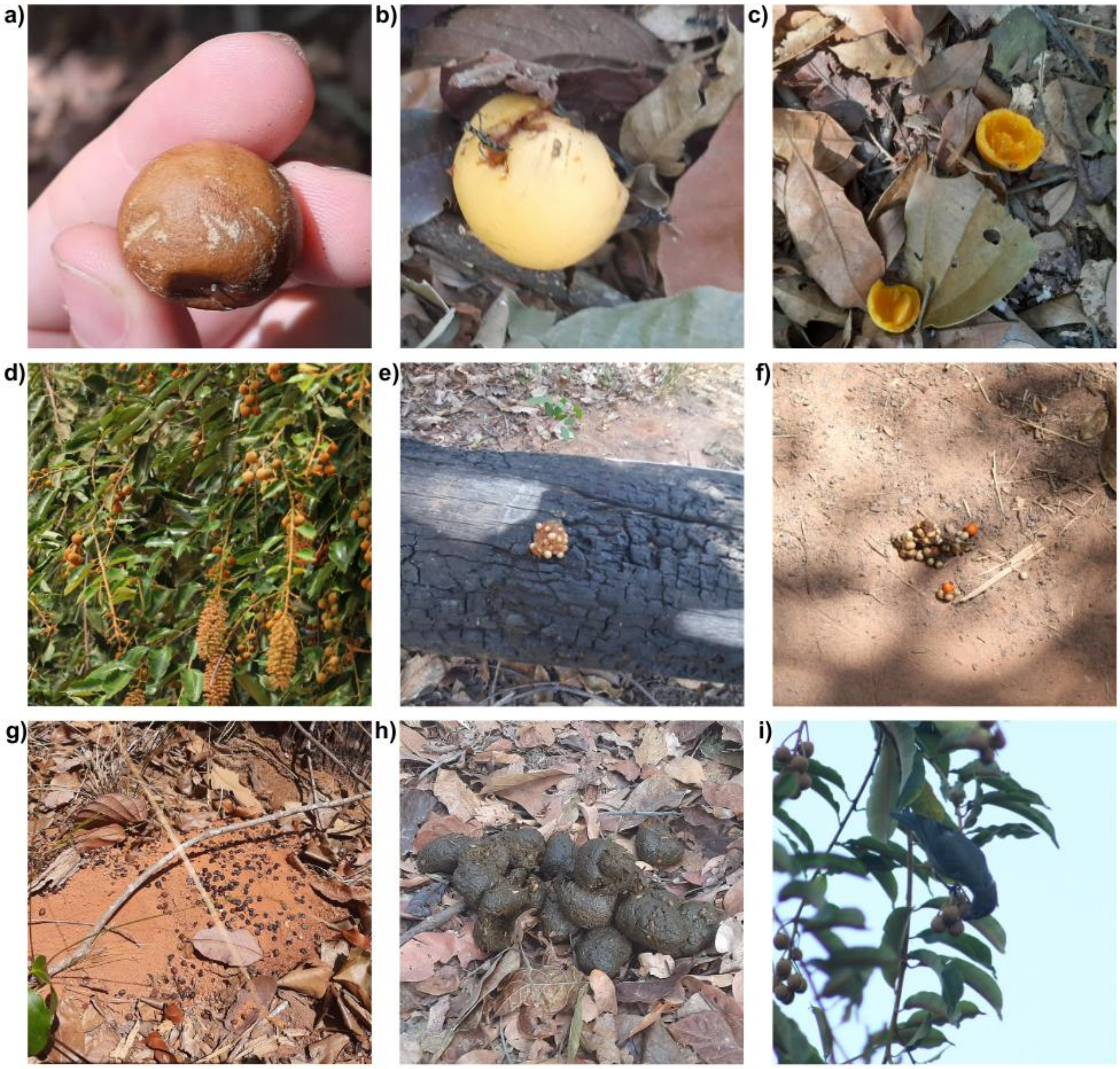

Fig. 5 Natural fruits and seed found among the sample areas with evidence of interactions with local fauna: **a)** fruit with marks of attacks carried out by birds; **b)** fruit being accessed by ants on the forest floor; **c)** fruit after being consumed by primates; **d)** fruits of the pioneer species *Mabea fistulifera* found in abundance on forest edges and burned areas; **e)** feces with the presence of seeds deposited in a burned area; **f)** feces with the presence of diaspores deposited forest ground; **g)** seeds of *M. fistulifera* and other species present around an ant nest; **h)** Tapir' feces with the presence of seeds; **i)** *Thraupis sayaca*, a low-quality disperser, in *M. fistulsifera* fruits

Table S1 Contribution rates of different animal groups interacting with artificial fruits between experiments (understory and terrestrial interactions). In bold is the total number of fruits manipulated by all fauna relative to the number of fruits offered per stratum across unburned interior, unburned edge, burned interior and burned edge treatments (800 fruits). The contribution rates of different faunal groups are expressed relative to the total number of fruits manipulated (i.e., interaction total rate)

Stratum	Taxa	Manipulated fruits	Contribution
Understory	**All taxa**	**212**	**26.5%**
	Birds	115	54.2%
	Mammals	29	13.6%
	Invertebrates	45	21.2%
	Indeterminate	29	13.67%
Terrestrial	**All taxa**	**236**	**29.5%**
	Birds	9	3.8%
	Mammals	52	22.03%
	Invertebrates	179	75.8%
	Indeterminate	4	1.69%

Table S2 Minimum, maximum, mean and standard error values of the proportion of manipulated fruits (understory and terrestrial) across unburned interior, unburned edge, burned interior, and burned edge treatments

Stratum	Treatments	Min	Max	Mean	SE
Understory	unburned interior	0	1	0.17	0.04
	unburned edge	0	1	0.33	0.06

	burned interior	0	1	0.33	0.07
	burned edge	0	1	0.23	0.06
Terrestrial	unburned interior	0	1	0.25	0.08
	unburned edge	0	1	0.49	0.09
	burned interior	0	1	0.19	0.05
	burned edge	0	1	0.23	0.08

Table S3 Results of contrast tests for the animal-fruit terrestrial interactions by pairwise comparisons across unburned interior, unburned edge, burned interior, and burned edge treatments. Highlighted p-values represent significant differences

Pairwise comparisons	Chi	df	p
unburned interior × unburned edge	3.82	1	**0.05***
unburned interior × burned interior	0.01	1	0.90
unburned interior × burned edge	0.40	1	0.52
unburned edge × burned interior	3.65	1	**0.05***
unburned edge × burned edge	6.60	1	**0.01***
burned edge × burned interior	0.59	1	0.43

Table S4 Rate of seed manipulation across the different full-fauna and invertebrates-only access. The overall rate of seed manipulation is shown in bold. Additionally, the rates of the types of manipulation observed in the experiments (seeds removed or predated) are presented relative to the total number of seeds manipulated (i.e., total animal–seed interactions)

Experiment	Interaction type	Manipulated seeds	Proportion

Full-fauna access	**Manipulated seeds**	**1074**	**89.5%**
	Removed seeds	1,015	94.5%
	Predated seeds	59	5.49%
Invertebrates-only access	**Manipulated seeds**	**1096**	**91.33%**
	Removed seeds	1,036	94.52%
	Predated seeds	60	5.47%

Table S5 Minimum, maximum, mean and standard error values of the proportion of manipulated seeds exposed on full-fauna access and on invertebrates-only access across unburned interior, unburned edge, burned interior and burned edge treatments

Experiment	Treatments	Min	Max	Mean	SE
Full-fauna access	unburned interior	0.3	1	0.96	0.02
	unburned edge	0.5	1	0.96	0.02
	burned interior	0.2	1	0.84	0.03
	burned edge	0.0	1	0.81	0.05
Invertebrates-only access	unburned interior	0.7	1	0.97	0.01
	unburned edge	0.4	1	0.95	0.02
	burned interior	0.4	1	0.92	0.02
	burned edge	0.2	1	0.80	0.04

Table S6 Results of contrast tests for the seed manipulated experiments by pairwise comparisons across forest treatments: unburned interior, unburned edge, burned interior and burned edge treatments. Highlighted p-values represent significant differences

Experiment	Pairwise comparisons	Chi	df	p
Full-fauna access	unburned interior × unburned Edge	0.13	1	0.71
	unburned interior × burned Interior	9.98	1	**0.001***
	unburned interior × burned edge	5.94	1	**0.01***
	unburned edge × burned interior	12.05	1	**< 0.001***
	unburned edge × burned edge	7.58	1	**0.005***
	burned edge × burned interior	0.39	1	0.52
Invertebrates-only access	unburned interior × unburned edge	0.53	1	0.46
	unburned interior × burned interior	3.91	1	**0.04***
	unburned interior × burned edge	8.44	1	**0.003***
	unburned edge × burned interior	1.5	1	0.21
	unburned edge × burned edge	4.96	1	**0.02***
	burned edge × burned interior	1.17	1	0.27
